# Identification and validation of a prognostic risk-scoring model based on sphingolipid metabolism-associated cluster in colon adenocarcinoma

**DOI:** 10.3389/fendo.2022.1045167

**Published:** 2022-11-28

**Authors:** Qihang Yuan, Weizhi Zhang, Weijia Shang

**Affiliations:** ^1^ Department of General Surgery, First Affiliated Hospital of Dalian Medical University, Dalian, Liaoning, China; ^2^ Laboratory of Integrative Medicine, First Affiliated Hospital of Dalian Medical University, Dalian, Liaoning, China; ^3^ Dalian No.24 High School, Dalian, Liaoning, China

**Keywords:** colon adenocarcinoma, sphingolipid metabolism, pan-cancer analysis, hierarchical clustering, stratification model, prognostic biomarker

## Abstract

Colon adenocarcinoma (COAD) is the primary factor responsible for cancer-related mortalities in western countries, and its development and progression are affected by altered sphingolipid metabolism. The current study aimed at investigating the effects of sphingolipid metabolism-related (SLP) genes on multiple human cancers, especially on COAD. We obtained 1287 SLP genes from the GeneCard and MsigDb databases along with the public transcriptome data and the related clinical information. The univariate Cox regression analysis suggested that 26 SLP genes were substantially related to the prognosis of COAD, and a majority of SLP genes served as the risk genes for the tumor, insinuating a potential pathogenic effect of SLP in COAD development. Pan-cancer characterization of SLP genes summarized their expression traits, mutation traits, and methylation levels. Subsequently, we focused on the thorough research of COAD. With the help of unsupervised clustering, 1008 COAD patients were successfully divided into two distinct subtypes (C1 and C2). C1 subtype is characterized by a poor prognosis, activation of SLP pathways, high expression of SLP genes, disordered carcinogenic pathways, and immune microenvironment. Based on the clusters of SLP, we developed and validated a novel prognostic model, consisting of ANO1, C2CD4A, EEF1A2, GRP, HEYL, IGF1, LAMA2, LSAMP, RBP1, and TCEAL2, to quantitatively evaluate the clinical outcomes of COAD. The Kaplain-Meier survival curves and ROC curves highlighted the accuracy of our SLP model in both internal and external cohorts. Compared to normal colon tissues, expression of C2CD4A was detected to be significantly higher in COAD; whereas, expression levels of EEF1A2, IGF1, and TCEAL2 were detected to be significantly lower in COAD. Overall, our research emphasized the pathogenic role of SLP in COAD and found that targeting SLP might help improve the clinical outcomes of COAD. The risk model based on SLP metabolism provided a new horizon for prognosis assessment and customized patient intervention.

## Introduction

Colon adenocarcinoma (COAD) is a type of malignant tumor originating from the colon gland epithelium and the most prevalent pathological form of colon tumor. Colorectal cancer is the fourth most prevailing malignant tumor in China, and its prevalence is increasing (1). The clinical manifestations of COAD differ based on the tumor’s location and stage. Due to the unique nature of the signs of early colon cancer, some individuals are diagnosed at an advanced stage. Currently, the majority of patients with colon cancer may benefit from a comprehensive treatment regimen that includes extensive surgery, radiation, and chemotherapy (2). However, the unfavorable effects of surgery, radiation, and chemotherapy will also contribute to a reduction in the quality of life of individuals suffering from colon cancer, and the prognosis for those who cannot withstand surgical treatment, radiotherapy, and chemotherapy remains grim. In recent years, the in-depth investigation of tumor metabolic reprogramming and immunological microenvironment has provided evidence that both metabolic and immune heterogeneity plays a role in the onset and progression of colon cancer (3). Patients having diverse metabolic features may have a distinct immune microenvironment and prognosis. Accurate identification of the metabolic heterogeneity of colon cancer is a crucial problem for the advancement of precise oncology therapy.

According to previous biological studies on sphingolipid metabolism and its function, ceramide, ceramide-1-phosphate, glucosylceramide, lactose ceramide, galactosylceramide, sphingosine, sphingosine galactoside, and 1-phosphate-sphingosine are not only inactive precursors of sphingomyelin metabolism but also crucial effector molecules in cell signal transduction (4). It has been shown that sphingolipids and their metabolites play a role in several crucial processes of signal transduction, such as cell growth, differentiation, senescence, and programmed cell death, resulting in a wide range of cellular biological activities (5). Since ceramide was discovered to be the second messenger of lipids to promote apoptosis, multiple studies have demonstrated that aberrant sphingolipid metabolism is strongly linked to the onset and progression of cancer; however, the exact mechanism remains unclear (6).

This study aimed at exploring the possible role of sphingolipid metabolism in pan-cancer, particularly colon cancer, and to provide a new scheme for the prognosis assessment as well as tailored patient therapy. In addition, the influence of sphingolipid metabolism on the tumor immune milieu was investigated further, laying the groundwork for the investigation of the link between metabolic reprogramming and the tumor immune microenvironment. Considering the substantial impact of sphingolipid metabolism on the survival and treatment options of COAD patients, we developed a unique and reliable prognostic discriminant model based on the sphingolipid metabolism classification technique.

## Material and methods

### Acquisition of COAD datasets and SLP genes

The gene expression profiles and relevant clinicopathological data from COAD patients were retrieved from the TCGA (https://portal.gdc.cancer.gov/) and GEO (https://www.ncbi.nlm.nih.gov/) datasets. The TCGA dataset consisting of 473 COAD samples was used as the training dataset, while the GSE39582 cohort containing 566 COAD samples was used as the validation dataset. The “sva” package (7) in R was implemented for generating the relative gene expression matrices from the TCGA and GSE39582 cohorts. Until now, over 1,200 genes related to sphingolipid metabolism have been discovered on the “GeneCards (8)” and “MSigDB (9)” platforms. These SLP genes were selected for further expression profile analyses.

### Identification of prognostic SLP genes

Univariate Cox regression analysis adjusted by the Benjamini & Hochberg (BH) technique was implemented to screen the prognostic SLP genes in both the TCGA and GSE39582 datasets (10). After the intersection, SLP genes with potential prognostic values in both datasets were obtained. In addition to the univariate Cox regression analysis, KM survival curves were also plotted for the purpose of verifying the prognostic performances of the above 26 SLP genes with the help of “survival” and “survminer” packages in R (11). Finally, the “cor” function in R was applied to explore the co-expression relationship between these 26 prognostic SLP genes in both the TCGA and GSE39582 datasets.

### The pan-cancer landscape of SLP genes

The raw data of pan-cancer comprising the mRNA expression profiles, clinical information, single nucleotide variation (SNV) data, copy number variation (CNV) data, and methylation data were gathered from The Cancer Genome Atlas (TCGA) (https://portal.gdc.cancer.gov/). The “limma” package in R was utilized for analyzing the differential expression of SLP genes in pan-cancer-related tumor tissues and paracancerous tissues.

TCGA database was used to collect the SNV data across 33 cancer types. The collected data include variant-type values: Missense_Mutation, Silent, 5’ Flank, 3’ UTR, RNA, In_Frame_Del, Nonsense_Mutation, SLPice_Site, Intron, 5’ UTR, In_Frame_Ins, Frame_Shift_Del, Nonstop_Mutation, 3’ Flank, Frame_Shift_Ins, and Translation_Start_Site. The Silent, Intron, IGR, 3’ UTR, 5’ UTR, 3’ Flank, and 5’ Flank were extracted for calculating the SNV percentage. SNV mutation frequency (percentage) of the coding region of each gene was computed using the formula: Number of Mutated Samples/Number of Cancer Samples. An SNV oncoplot plot was produced by maftools (12). Moreover, each gene’s CNV was elevated by incorporating the heterozygosity and homozygosity of amplification and deletion, in which over 5% was considered to be a high-frequency CNV. The R language was applied to visualize the results of SNV and CNV of SLP genes in pan-cancer. The methylation probe for the individual gene’s promoter was annotated using the R package “IlluminaHumanMethylation-450kanno.ilmn12.hg19” from Bioconductor. The Wilcoxon signed rank test was utilized for examining the differential methylation between all genes in the tumor as well as healthy samples, and a P-value cutoff of 0.05 was used for identifying the genes that were substantially hypo-methylated or hyper-methylated. Furthermore, single sample gene set enrichment analysis (ssGSEA) was employed for computing SLP scores in individual tumor samples so as to identify the pathways related to sphingolipid metabolism in several human malignancies (13). In addition, based on the SLP scores, the samples of every tumor type were classified into two groups, comprising the top 30% and the bottom 30%. Gene set enrichment analysis (GSEA) was then carried out similarly to our previous studies (14, 15).

### SLP-based cluster analysis

A total of 1008 COAD patients were clustered into two subgroups following the SLP-related gene expression profiles using the R software package “ConsensusClusterPlus” (16). The highest number of clusters was 9, and 80% of the total samples were drawn 50 times, clusterAlg = “km”, distance = “euclidean”. With the aid of R software packages “survival” and “survminer”, the Kaplan-Meier survival analysis was then performed for the purpose of comparing the survival difference between the two groups. Moreover, the Wilcoxon test was conducted to observe the expression distribution of the SLP genes in both subtypes related to SLP (17). Furthermore, 9 typical SLP-associated pathways and 50 typical cancer-associated pathways were downloaded from the MSigDb platform (9). The “GSVA” package and the “Wilcox.test” function in R were employed to assess and compare the activities of the aforementioned pathways between the two subtypes (18).

### Cluster-based analysis of tumor immune microenvironment

Sphingolipids are implicated in the interactions between cancer cells and immune system, as shown by a rising number of studies. In order to characterize the discrepancy in the immune response between C1 and C2 subtypes at the macroscopic level, the R program “estimate” was first used for calculating the immune score, stromal score, tumor purity, and estimate the score of individual COAD samples (19). The “estimate” algorithm was developed by Kosuke et al (20). Based on the expression data, the “estimate” method could estimate the matrix percentage (stromalscore) and immune score (immunescore) of tumour samples, which might be used to indicate the presence of matrix and immune cells. Adding the two fractions yields the estimatescore, which might be used to estimate the tumor’s purity. Subsequently, the R package “ggpubr” was utilized for visualizing the results. In order to uncover the infiltration abundance of each type of immunocytes, the CIBERSORT, MCPcounter, QUANTISEQ, XCELL, CIBERSORT-ABS, TIMER, and EPIC algorithms were employed to assess the immunological characteristics of both subtypes (21). Above 7 immunological algorithms were conducted with the help of TIMER2.0 platform, which was a public website using the immunedeconv method to assess the abundance of varied immunocyte infiltration. Subsequently, a heatmap was plotted to visualize the results at the microscopic level. Lastly, we studied the expression of common immune checkpoint genes (ICGs) across various subtypes for predicting the efficacy of immune checkpoint blockade therapy (22).

### Cluster-based prediction of drug sensitivity

For estimating the sensitivity of chemotherapeutic agents, the R package pRRophetic was employed for determining the half-maximal inhibitory concentration (IC50) of samples in various groups *via* ridge regression (23). The pRRophetic package is a program developed by Prof. Paul Geeleher of the University of Minnesota (23, 24). The basic principle of this algorithm is based on the expression profile of the cell line and the corresponding IC50 information, and then establish a model through ridge regression, and then use this model to predict the chemotherapeutic response of clinical samples. Subsequently, the IC50 values across various subtypes were compared using the Wilcoxon test. A lower IC50 value indicated a better response to chemotherapy.

### Cluster-based annotation of differentially expressed genes

The “limma” R package was utilized for screening DEGs across several clusters (|logFC| > 0.585, FDR < 0.05) (25). Gene Ontology (GO) enrichment analysis and Kyoto Encyclopedia of Genes and Genomes (KEGG) analysis were carried out for selecting and visualizing the significantly enriched GO terms and KEGG pathways in DEGs (26–28).

### DEGs-based development and verification of a novel SLP-associated prognostic panel

The 446 samples of COAD in the TCGA data set were sorted into training and validation cohorts. All the samples were placed back into the random grouping 100 times in advance to prevent the impact of random assignment bias on the stability of subsequent modeling, and the group sampling was done with a training cohort-to-validation cohort ratio of 1:1. Moreover, 224 samples were present in the final training cohort and 222 samples in the final internal validation cohort 1 (i.e. test1). All the 446 samples of COAD in the TCGA data set were rated as the internal validation cohort 2 (i.e., test2). Moreover, 562 COAD samples in the GSE39582 data set were rated as the external validation cohort (i.e., test 3). For DEGs between different molecular subtypes, the univariate Cox regression analysis was carried out using the coxph function of the survival package in R, and a P-value of < 0.05 was taken as the filtering threshold. Moreover, Lasso regression using R package glmnet was carried out for the purpose of eliminating the redundancy of prognostic genes for subsequently developing the prognostic model (29). Finally, multivariate Cox regression analysis and the “predict” function in R were employed for computing the risk scores of every sample of COAD. The samples in the training, test1, test2, and test3 were all allocated into high and low-risk subpopulations following the median risk score of the training cohort. KM survival curves and ROC curves were drawn for evaluating as well as calculating the prognostic values of SLP-APP in the aforementioned cohorts.

### Differential expression analysis of SLP-APP genes

A multidimensional cancer genomics dataset called GEPIA incorporated mass data from The Cancer Genome Atlas (TCGA) and the Genotype-Tissue Expression project (GTEx) (30, 31). Boxplot could be employed to determine the expression level of a single gene in various cancer types. We identified the expression levels of SLP-APP genes in COAD as well as healthy colon tissues based on TCGA and GTEx data. Human Protein Atlas (HPA, https://www.proteinatlas.org/) was used for determining the protein expression in different tissues and organs of humans from the RNA and protein levels by using transcriptomics and proteomics techniques (32, 33). We identified the SLP-APP protein expression in both COAD and healthy colon tissues based on HPA data.

### Functional analysis of SLP metabolism-associated genes in COAD based on the knock-down of HIF1α gene

On the GEO platform, we discovered knock-down sequencing data for the HIF1a gene after a search of more than 1200 sphingolipid metabolism genes previously compiled. Consequently, we investigated the function and possible mechanism of HIF1α in COAD. GSE155104 is an open cell line knock-down sequencing data set submitted by Prof.Glaus Garzon JF et al (34). The authors cultivated the mouse colon cancer cell line MC38 and silenced the HIF1α gene. The transcriptomes of control cells and knock-down cell lines were sequenced. We gathered and compiled these sequencing data, examined the differentially expressed genes of the two cell lines using the limma programme, and assessed the enrichment of the GO and KEGG ontologies.

## Results

### Identification of prognosis-related SLP genes

**Figure 1:**
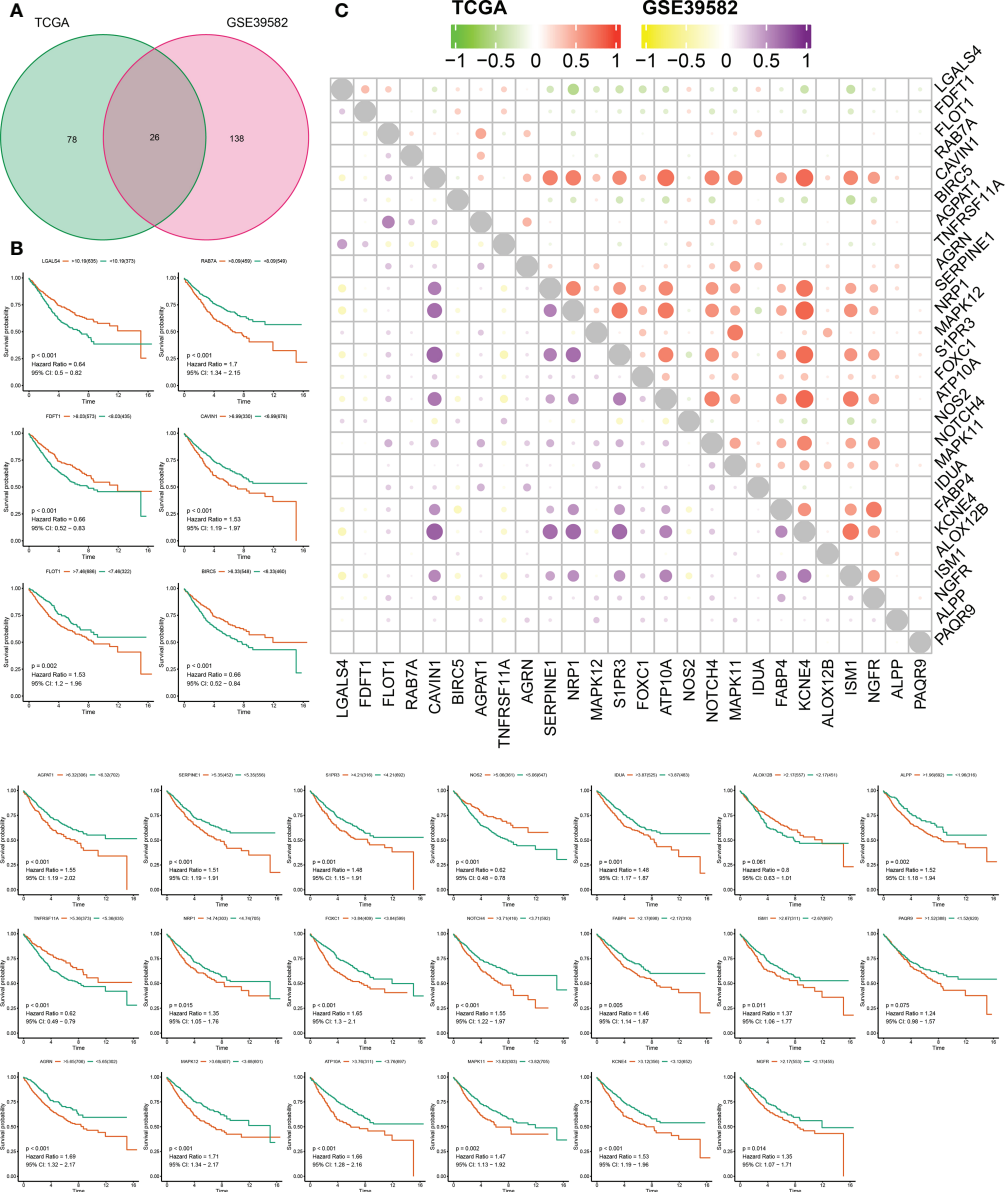
Prognostic performances of SLP genes in colon adenocarcinoma. **(A)** The results of univariate Cox regression analysis: Green represents 104 genes obtained by TCGA screening, red represents 164 genes obtained by geo screening, and 26 SLP genes obtained by intersection; **(B)** KM survival curves of 26 SLP genes; **(C)** Co-expression relationship between these 26 SLP genes in both TCGA and GEO cohorts.

Using the “GeneCards” and “MSigDB” databases, 1287 SLP genes were detected (**Table S1**). It was found that 104 SLP genes were significantly linked to COAD prognosis using univariate Cox analysis in the TCGA cohort (**Table S2**). Similarly, 164 SLP genes were also significantly associated with the prognosis of COAD according to the univariate Cox analysis in the GSE39582 cohort (**Table S3**). After the intersection, 26 SLP genes were finally preserved as reliable SLP genes related to prognosis (**Figure 1A**). KM survival curves of 26 SLP genes were constructed to verify their prognostic performances (**Figure 1B**). The co-expression relationship of 26 genes was shown in **Figure 1C**. It was found that KCNE4 and CAVIN1 were positively correlated with most genes, such as NRP1 and S1PR3. These 26 SLP genes included LGALS4, FDFT1, FLOT1, RAB7A, CAVIN1, BIRC5, AGPAT1, TNFRSF11A, AGRN, SERPINE1, NRP1, MAPK12, S1PR3, FOXC1, ATP10A, NOS2, NOTCH4, MAPK11, IDUA, FABP4, KCNE4, ALOX12B, ISM1, NGFR, ALPP, PAQR9. As shown in **Table 1** (35–56), the majority of genes may operate as proto-oncogenes and affect the cell proliferation, invasion, migration, and metastasis of COAD patients.

**Table 1:** Contributions of 26 sphingolipid metabolism-related genes in COAD.

Gene Symbol	Expression Level^a^	Role^b^	Functional Phenotype	Molecular Mechanism	References
LGALS4	Down-regulation	TSG	cell proliferation	IL-6/NF-κB/STAT3 signaling pathway	Kim et al. (35)
FDFT1	Up-regulation	POG	cell proliferation tumor growth	NAT8 and D-pantethine	Jiang et al. (36)
FLOT1	Up-regulation	POG	cell proliferation	Unknown	Baig et al. (37)
RAB7A	Unknown	Unknown	Unknown	Unknown	Unknown
CAVIN1	Down-regulation	Unknown	Unknown	Unknown	Unknown
BIRC5	Up-regulation	Unknown	cell viability apoptosis induction	BMF and Eomesodermin	Wang et al. (38)
AGPAT1	Up-regulation	POG	Unknown	Unknown	Karagiota et al. (39)
TNFRSF11A	Up-regulation	POG	cell migration, invasion, and metastasis	Ca2+-calcineurin/NFATC1-ACP5 axis	Liang et al. (40)
AGRN	Up-regulation	Unknown	Unknown	Unknown	Unknown
SERPINE1	Up-regulation	POG	cell migration cell death	MMP1	Kim et al. (41)
NRP1	Up-regulation	POG	endothelial cell migration angiogenesis	EGF and mitogen-activated protein kinase signaling pathways	Parikh et al. (42)
MAPK12	Up-regulation	POG	inflammation	β-catenin/Wnt activities	Yin et al. (43)
S1PR3	Up-regulation	POG	Cell proliferation migration invasion apoptosis	Unknown	Grbčić et al. (44)
FOXC1	Up-regulation	POG	Cell growth metastasis	SNAIL1/epithelial-to-mesenchymal transition	Li et al. (45)
ATP10A	Up-regulation	Unknown	Unknown	Unknown	Unknown
NOS2	Up-regulation	POG	invasiveness metastasis apoptosis autophagy	NO/nitrosative stress	Castro et al. (46) Spiegel et al. (47)
NOTCH4	Down-regulation	TSG	cell proliferation migration invasion apoptosis	NOTCH4-GATA4-IRG1 axis	Scheurlen et al. (48) Wu et al. (49) Zhang et al. (50)
MAPK11	Down-regulation	Unknown	Unknown	T-type Ca (2+) channel/CDKN1A/BBC3/PUMA	Dziegielewska(51)
IDUA	Down-regulation	Unknown	Unknown	Unknown	Unknown
FABP4	Up-regulation	POG	Cell invasion metastasis	fatty acid transport	Tian et al. (52)
KCNE4	Up-regulation	POG	Unknown	Unknown	Liu et al. (53)
ALOX12B	Unknown	Unknown	Unknown	Unknown	Unknown
ISM1	Up-regulation	POG	cell migration proliferation	epithelial-mesenchymal transition	Wu et al. (54)
NGFR	Down-regulation	TSG	cell proliferation invasion colony formation apoptosis chemosensitivity	S100A9	Yang et al. (55) Chen et al. (56)
ALPP	Unknown	Unknown	Unknown	Unknown	Unknown
PAQR9	Unknown	Unknown	Unknown	Unknown	Unknown

Expression Level^a^: The increased expression level of genes in tumour tissues relative to normal or precancerous tissues is termed up-regulation, while the reduced expression level of genes in tumour tissues is termed down-regulation. Of note, this result was derived from the matched literatures or GEPIA2 platform (http://gepia2.cancer-pku.cn/#analysis)

Role^b^: TSG: tumor suppressor gene; POG: proto-oncogene.

### The pan-cancer landscape of SLP genes

**Figure 2:**
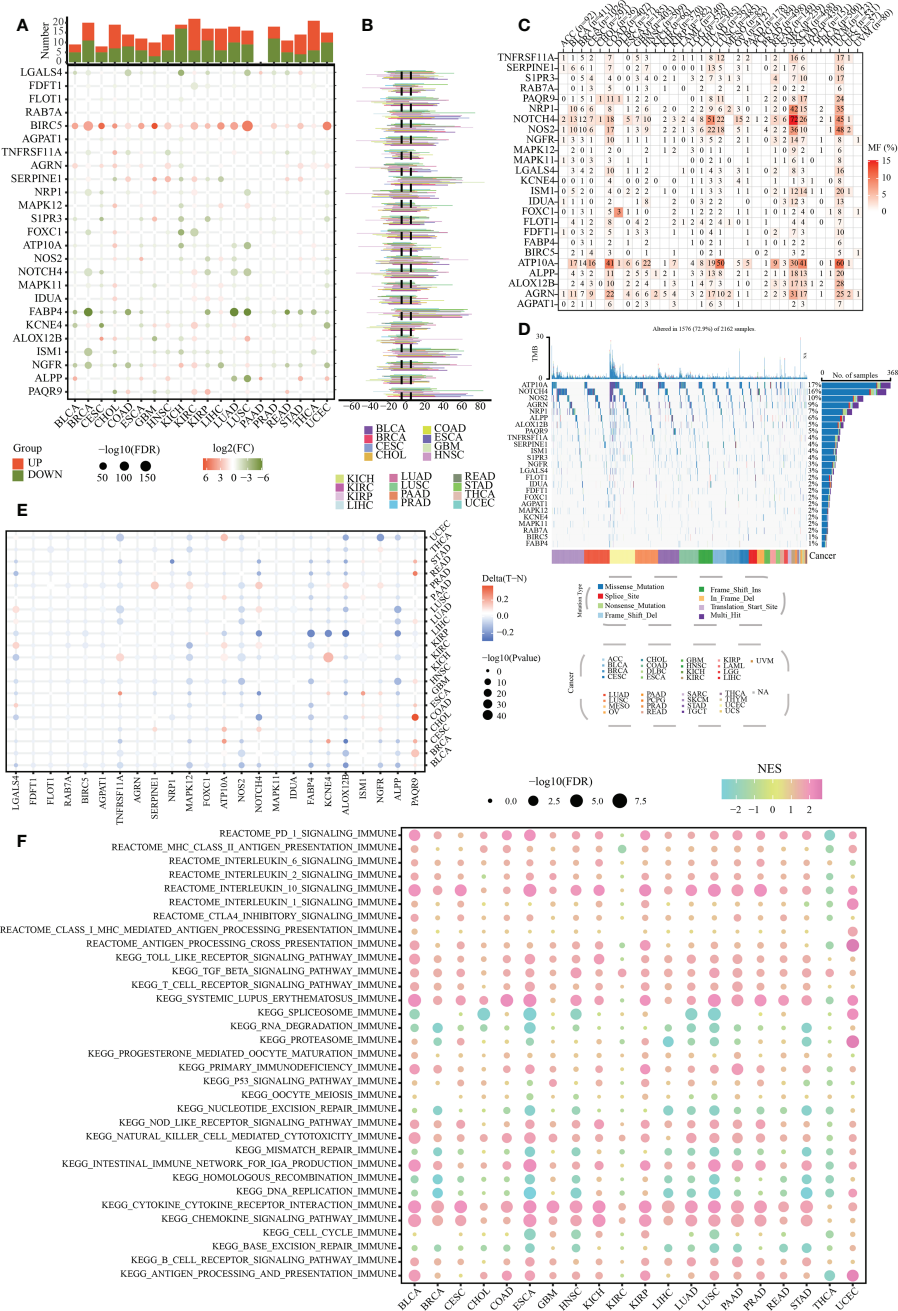
Pan-cancer overview of SLP genes. **(A)** Expression levels of 26 SLP genes in pan-cancer: Orange represents upregulation, green represents downregulation, and the circle size shows the degree of difference; **(B)** Copy number variation status of 26 SLP genes in pan-cancer; **(C)** Single nucleotide variant frequency of 26 SLP genes in pan-cancer; **(D)** Single nucleotide variant types of 26 SLP genes in pan-cancer; **(E)** The methylation levels of 26 SLP genes in pan-cancer; **(F)** Relationship of 26 SLP genes with immune pathways of pan-cancer.

To show the pan-cancer overview of the above-mentioned 26 SLP genes and reveal their potential biological function in cancers, we comprehensively investigated the mRNA expression traits, mutation landscapes, and methylation levels of these genes in multiple human cancers. Our results showed that BIRC5 was up-regulated, whereas FABP4 was down-regulated in most cancers (**Figure 2A**). Increased expression of BIRC5, SERPINE1, and PAQR9 was detected in COAD compared to that in the paracancerous tissues. Decreased expression of LGALS4, TNFRSF11A, FABP4, and NGFR was detected in COAD compared to that in the paracancerous tissues (**Figure 2A**). As depicted in **Figure 2B**, the CNVs of RAB7A, BIRC5, SERPINE1, and FABP4 were relatively high in most cancers. Significant SNV mutation of SLP genes (especially for NOTCH4 and ATP10A) was observed in COAD, LUAD, SKCM, and UCEC (**Figures 2C, D**). High methylation levels of PAQR9 were observed in most cancers, especially in the COAD (**Figure 2E**). Nevertheless, NOS2 exhibited a relatively low methylation level in most cancers. More importantly, these SLP genes were significantly linked to many typical immune-related pathways, including PD-1, IL-10, and chemokine signaling pathways (**Figure 2F**). Overall, these findings suggested a close association between SLP metabolism and tumor immune microenvironment.

### SLP-based cluster analysis

**Figure 3:**
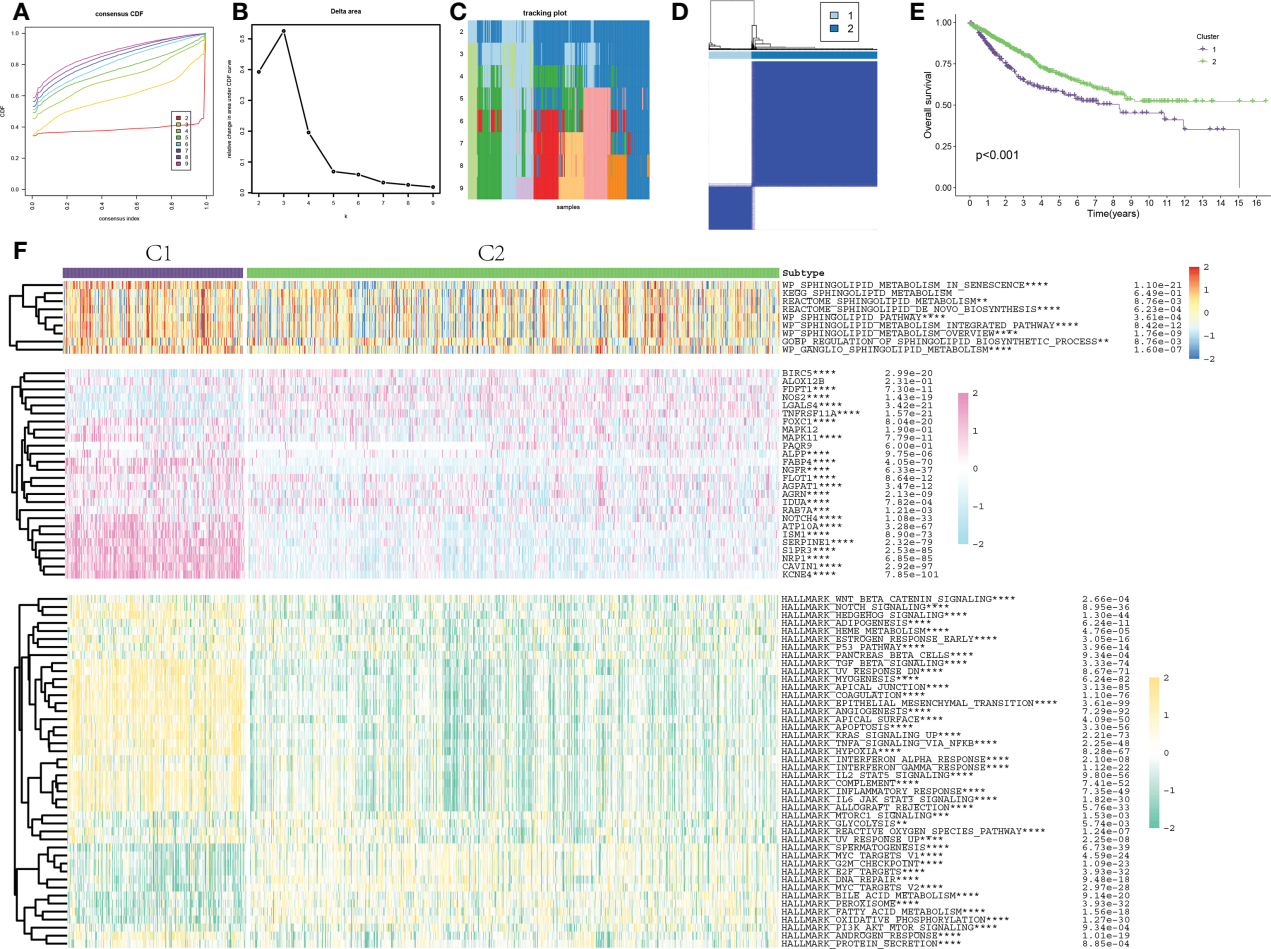
Consensus clustering and molecular characterization of colon adenocarcinoma. **(A, B)** Results of hierarchical clustering analysis show that the difference between clusters is the largest when k = 2; **(C)** When k = 2, the interference between clusters is minimal; **(D)** The cohort was classified into C1 and C2 clusters; **(E)** KM survival curve of two clusters: Purple represents C1 and green represents C2; **(F)** Heat map results of 9 typical SLP-related pathway activation, 26 SLP gene expression and 50 typical cancer-related pathway activation between the two subtypes. ** indicated p < 0.01, *** indicated p < 0.001, **** indicated p < 0.0001.

The 1008 samples of COAD could be classified into various clusters by following the consensus of mRNA expression of SLP genes. When the value of the clustering index “k” increased from 2 to 9, k = 2 was shown as the optimal point for attaining the largest differences (variations) between clusters (**Figures 3A, B**). Moreover, the interference between clusters was the least when k = 2 (**Figures 3C, D**). The COAD cohort was then sorted into cluster 1 (n = 255) and cluster 2 (n = 753) (**Figure 3D**). KM survival curves of the clusters indicated that C2-like COAD patients had higher rates of survival when compared to the C1-like COAD patients (p < 0.001, **Figure 3E**). The expression levels and pathway activities of SLP metabolism were substantially different between the two subtypes (**Figure 3F**). Most SLP genes were up-regulated in the C1 subtype. Meanwhile, the activities of most SLP pathways were also significantly increased in the C1 subtype. These findings indicated the activation of SLP metabolism might promote the progression and degree of COAD malignancy, thus contributing to the poor prognosis. Furthermore, the activities of typical cancer-related pathways exhibited remarkable differences between the C1 and C2 subtypes (**Figure 3F**). Specifically, the NOTCH signaling pathway, TGF-β signaling pathway, apoptosis, angiogenesis, and hypoxia pathways were significantly activated in the C1 subtype, which also might be responsible for the poor prognosis of the C1 subtype.

### Comparison of the immune landscape between two clusters

**Figure 4:**
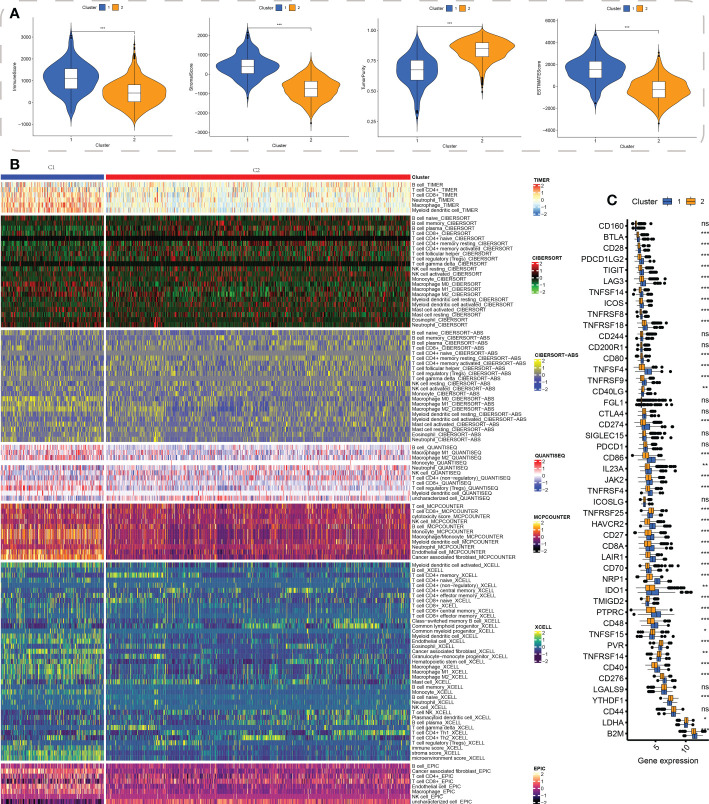
Cluster-based tumor immune microenvironment analysis. **(A)** ESTIMATE algorithm-derived immune-related scores between the two clusters, including stromal score, immune score, tumor purity, and estimate score; **(B)** The distribution of infiltration of the cells involved in the immune system between the clusters using a variety of immune algorithms, such as TIMER, CIBERSOFT, CIBERSOFT-ABS, QUANTISEQ, MCPCOUNTER, XCELL, and EPIC; **(C)** The common immune checkpoint genes’ expression levels in both the subgroups. * indicated p < 0.05, ** indicated p < 0.01, *** indicated p < 0.001, ns indicated not significance.

First, the R “estimate” package was employed for investigating the immune-related scores between the two subtypes, and these scores including stromal, immune, and estimate scores were substantially higher in the C1 subgroup (**Figure 4A**). To elucidate the infiltration distribution of each type of immunocytes between C1 and C2 subtypes, multiple deconvolution algorithms, including TIMER, CIBERSOFT, MCPCOUNTER QUANTISEQ, and EPIC, were performed. Similarly, the results demonstrated a higher abundance of B cells, CD4+ T cells, CD8+ T cells, and macrophages in C1 than in C2 (**Figure 4B**). Moreover, the expression levels of common genes of immune checkpoint were then compared between both subgroups; and the results showed the up-regulation of most of these genes (including PDCD1LG2, TIGIT, TNFSF4, LAG3, CD86, CD40, and CD48) in C1 (**Figure 4C**).

### Comparison of the drug sensitivity between the subgroups

**Figure 5:**
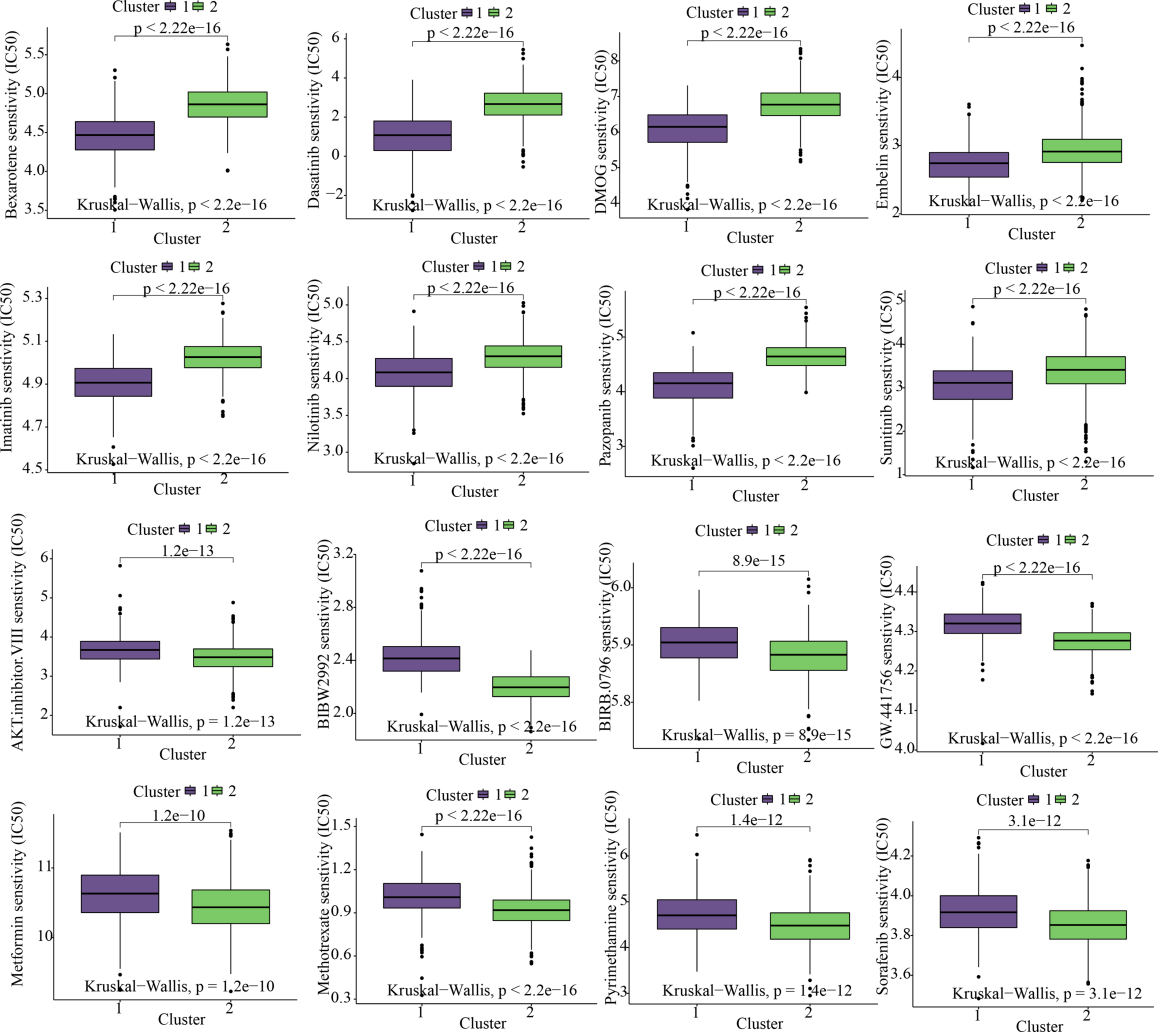
Cluster-based targeted-drug sensitivity analysis.

While taking into account the significant contribution of molecularly targeted therapy to the improvement of prognosis among individuals suffering from COAD, we evaluated the expression profile characteristics of various clusters by using the R package “pRRophetic” so as to find sensitive targeted therapeutic agents for C1 and C2 subtypes. The findings of our study revealed that the C1 subtype might gain benefit from Bexarotene, Dasatinib, DMOG, Embelin, Imatinib, Nilotinib, Pazopanib, and Sunitinib; however, the C2 subtype might benefit from AKT.inhibitor.VIII, BIBW2992, BIRB.0796, GW.441756, Metformin, Methotrexate, Pyrimethamine, and Sorafenib (**Figure 5**).

### Identification of differentially expressed genes and functional enrichment analysis

**Figure 6:**
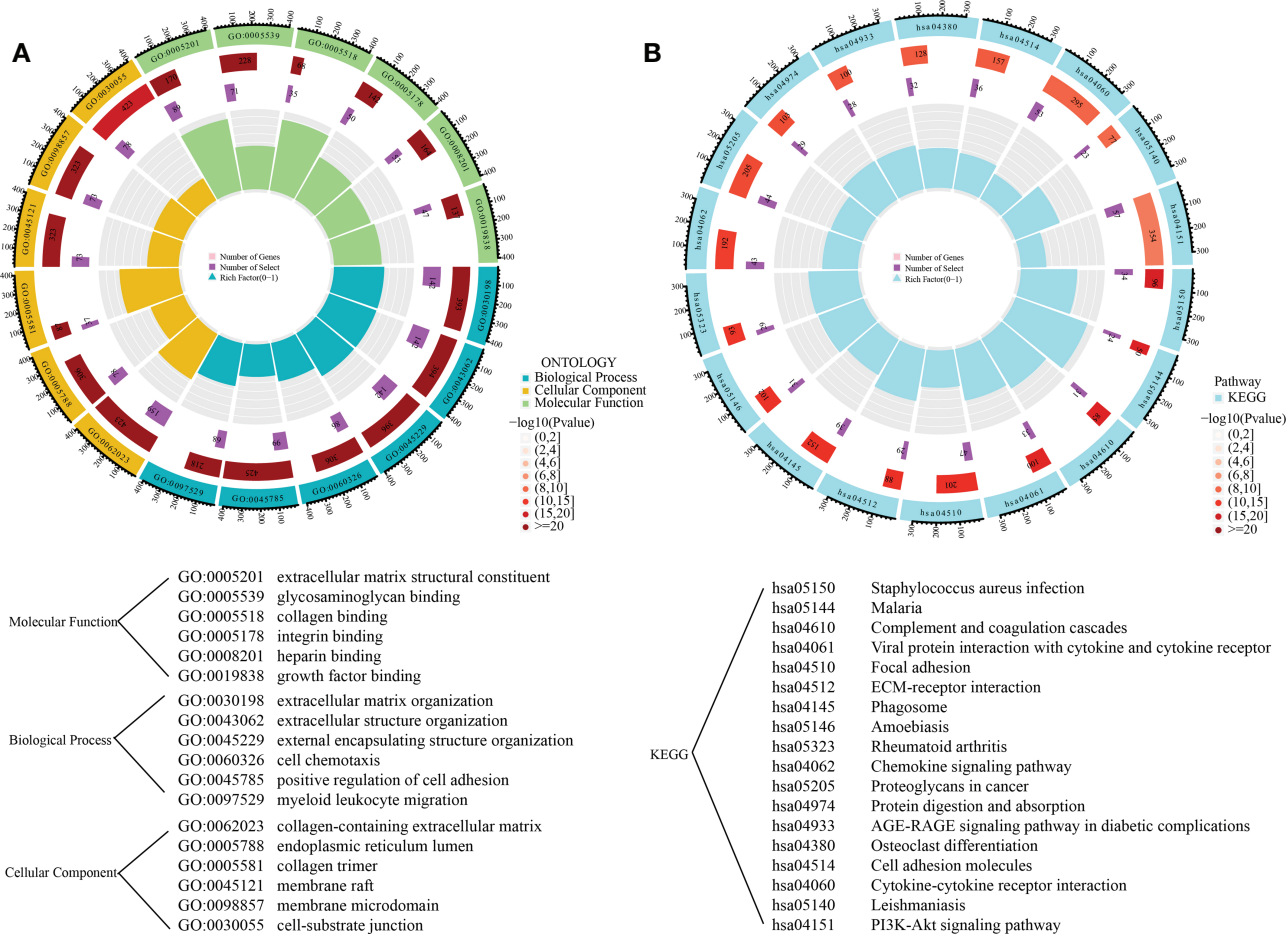
Functional enrichment analysis of DEGs between the subgroups. **(A)** The gene ontology enrichment results; **(B)** The KEGG enrichment results.

To explore the variations of biological functions between the clusters, 1381 DEGs between these clusters were detected. GO analyses indicated that DEGs were primarily enriched in extracellular matrix structural constituents, glycosaminoglycan binding, collagen binding, integrin binding, growth factor binding, and regulation of cell adhesion (**Figure 6A**). KEGG analysis demonstrated a primary involvement of DEGs in complement as well as coagulation cascades, focal adhesion, ECM-receptor interaction, as well as in chemokine and PI3K-Akt signaling pathways (**Figure 6B**).

### Identification and verification of SLP-associated prognostic panel

446 and 562 COAD samples retrieved from the TCGA database and the GEO database, respectively (all containing entire mRNA expression and survival data) were utilized for sorting the samples into various cohorts. The current study consisted of four cohorts: the training cohort (224 TCGA samples, making up 50% of the total), test1 cohort (222 TCGA samples, making up 50% of the total), test2 (all 446 TCGA samples), and test3 cohort (562 GEO samples). Particularly during the validation of SLP-APP, the test1 and test2 cohorts were employed for the internal, whereas, the test3 cohort was employed for the external validation.

**Figure 7:**
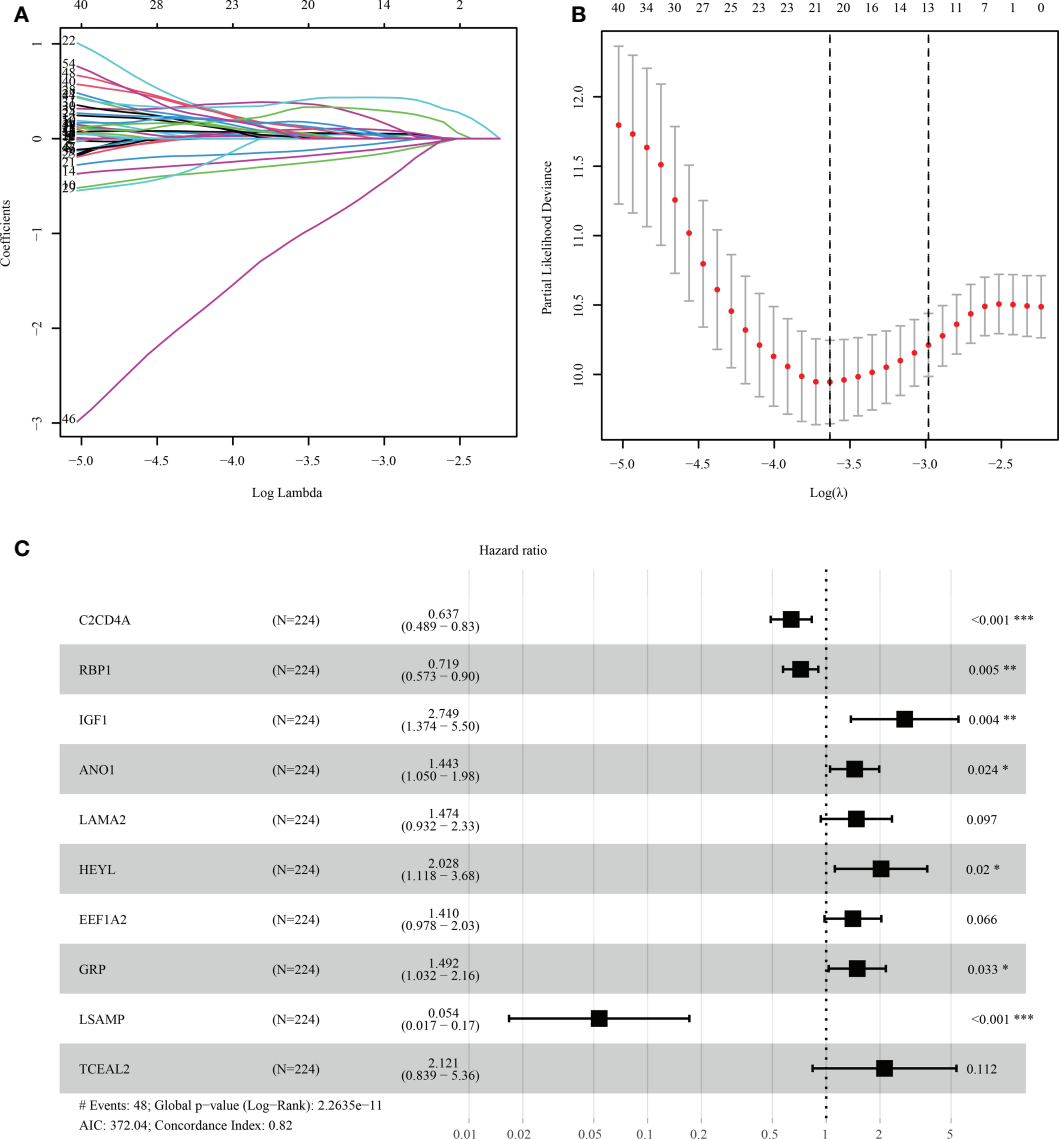
The process of LASSO-Cox regression analysis. **(A, B)** Lasso regression was performed to filter the candidate genes. **(C)** Multivariate Cox regression analysis aids in developing the prognostic model. * indicated p < 0.05, ** indicated p < 0.01, *** indicated p < 0.001

**Figure 8:**
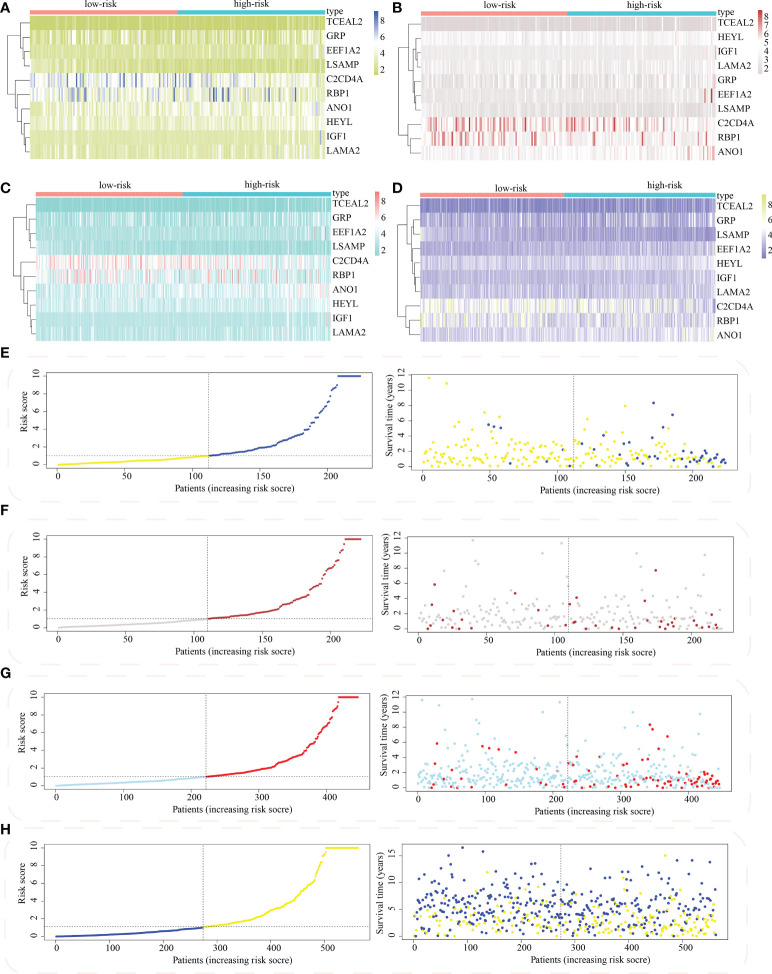
Distribution of model gene expression and patients’ survival between the subgroups with high and low risk. The model gene’s expression levels between subpopulations with high as well as low risk in **(A)** train, **(B)** test1, **(C)** test2, and **(D)** test3 cohorts. The survival status of colon adenocarcinoma between subpopulations of high and low risk in **(E)** train, **(F)** test1, **(G)** test2, and **(H)** test3 cohorts.

**Figure 9:**
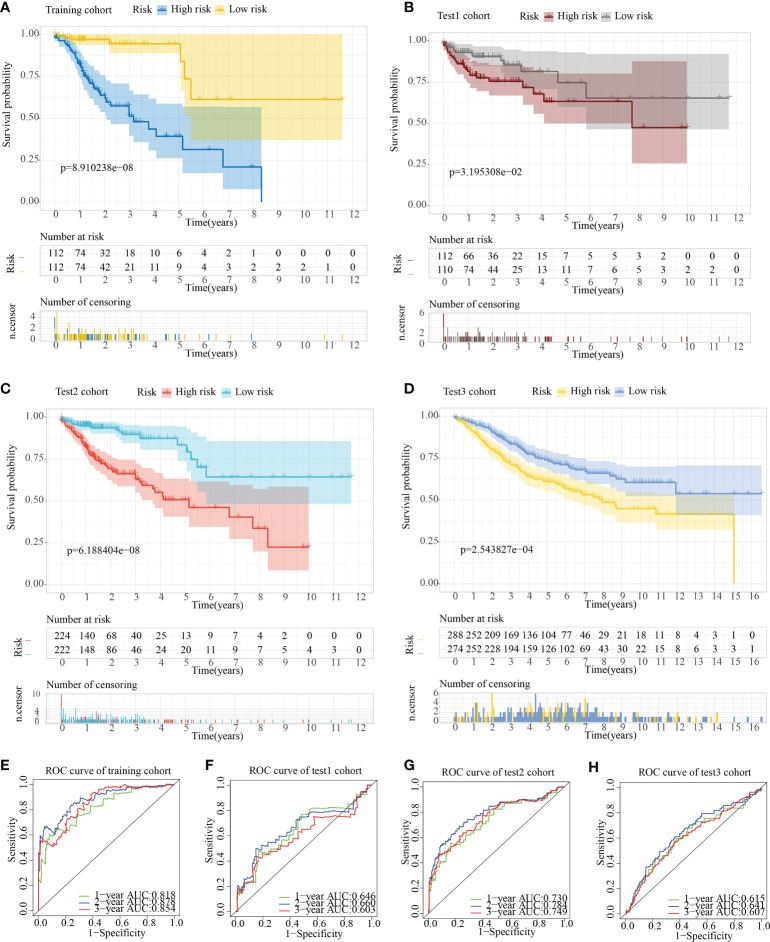
Assessment of the predictive ability of the SLP cluster-based prognostic model. Survival curve plots in **(A)** train, **(B)** test1, **(C)** test2, and **(D)** test3 cohorts. Receiver operating characteristic curves in **(E)** train, **(F)** test1, **(G)** test2, and **(H)** test3 cohorts.

The risk score model was initially constructed as per the training cohort. Univariate Cox regression analysis carried out on every DEG indicated that 55 of the 1381 DEGs were potential prognostic indicators (**Tables S4**, **S5**). Subsequently, LASSO regression analysis was carried out for eliminating collinearity among the 55 candidate genes and to prevent over-fitting of the prognostic model (**Figures 7A, B**). Finally, ten genes (i.e., TCEAL2, GRP, EEF1A2, LSAMP, C2CD4A, RBP1, ANO1, HEYL, IGF1, and LAMA2) were utilized in a multivariate Cox proportional hazards regression analysis for establishing a novel SLP-APP (**Figure 7C**). The risk scores on the basis of the risk model were calculated by the “predict” function in R, and the samples were categorized into high- and low-risk groups having the median risk score as the cut-off value. The levels of the 10 signature genes were then examined in the two groups (**Figures 8A–D**). The samples’ risk scores were ranked from low to high, and a scatter plot was used to visualize the survival status. According to the findings, an increase in risk scores led to a rise in patient fatalities and a decrease in their duration of survival (**Figures 8E–H**). The survival curves of training, test1, test2, and test3 cohorts displayed a remarkably lower length of survival in the group with a high risk as compared to the group with a low risk (**Figures 9A–D**). Moreover, the ROC curve indicated that, in the training dataset, AUC values for one-, two- and three-year OS were 0.818, 0.878, and 0.854, respectively (**Figure 9E**). In the test1 dataset, AUC values for one-, two- and three-year OS were 0.646, 0.660, and 0.603, respectively (**Figure 9F**). In the test2 dataset, AUC values for one-, two- and three-year OS were 0.730, 0.784, and 0.749, respectively (**Figure 9G**). In the test3 dataset, AUC values for one-, two- and three-year OS were 0.615, 0.641, and 0.607, respectively (**Figure 9H**). Overall, these findings verified that the 10-gene signature showed a satisfactory prognostic prediction effect.

### Differential expression analysis of SLP-APP genes

**Figure 10:**
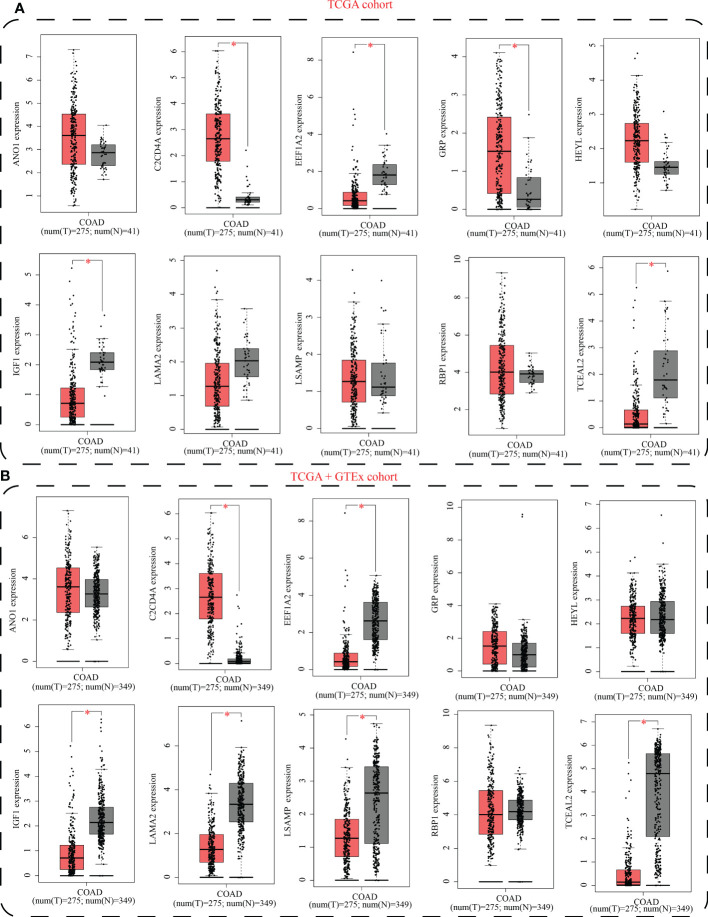
mRNA expression levels of model genes between colon adenocarcinoma and paracancerous/normal tissues using **(A)** TCGA cohort and **(B)** TCGA+GTEx cohorts. * indicated p < 0.05.

**Figure 11:**
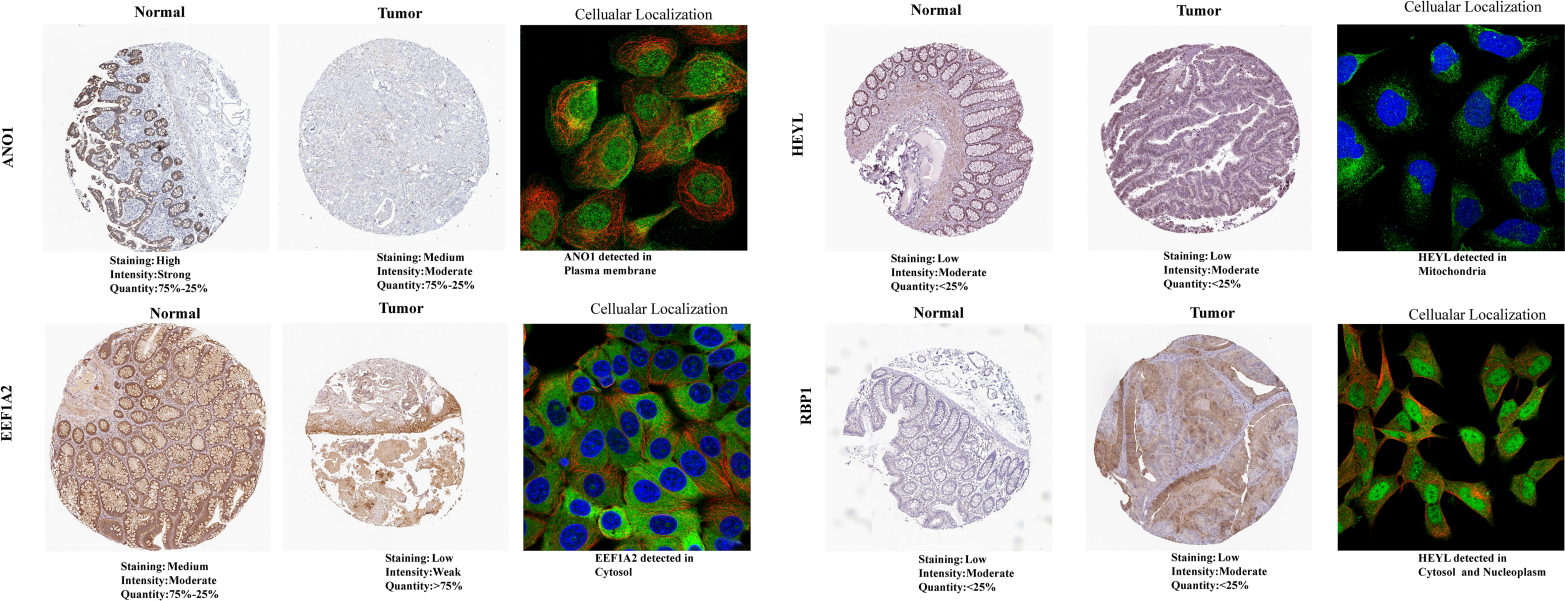
Protein expression levels and cellular location of model genes between colon adenocarcinoma and paracancerous/normal tissues using the HPA database.

The GEPIA2 database was utilized for comparing the mRNA expression levels of SLP-APP genes between cancer and paracancerous tissues. In the TCGA cohort, the transcriptomic levels of C2CD4A and GRP were substantially higher in COAD samples than in paracancerous tissues; nevertheless, the transcriptomic levels of EEF1A2, IGF1, and TCEAL2 were substantially lower in COAD samples than in paracancerous tissues (**Figure 10A**). In the TCGA and GTEx cohorts, the transcriptomic level of C2CD4A was substantially higher in COAD samples as compared to the paracancerous tissues; however, the transcriptomic levels of EEF1A2, IGF1, LAMA2, LSAMP, and TCEAL2 were significantly lower in COAD samples than paracancerous tissues (**Figure 10B**). The protein expression levels and cell location of SLP-APP genes in both cancer and adjacent tissues were observed using the HPA database (**Figure 11**).

### Functional analysis of SLP metabolism-associated genes in COAD based on the knock-down of HIF1α gene

**Figure 12:**
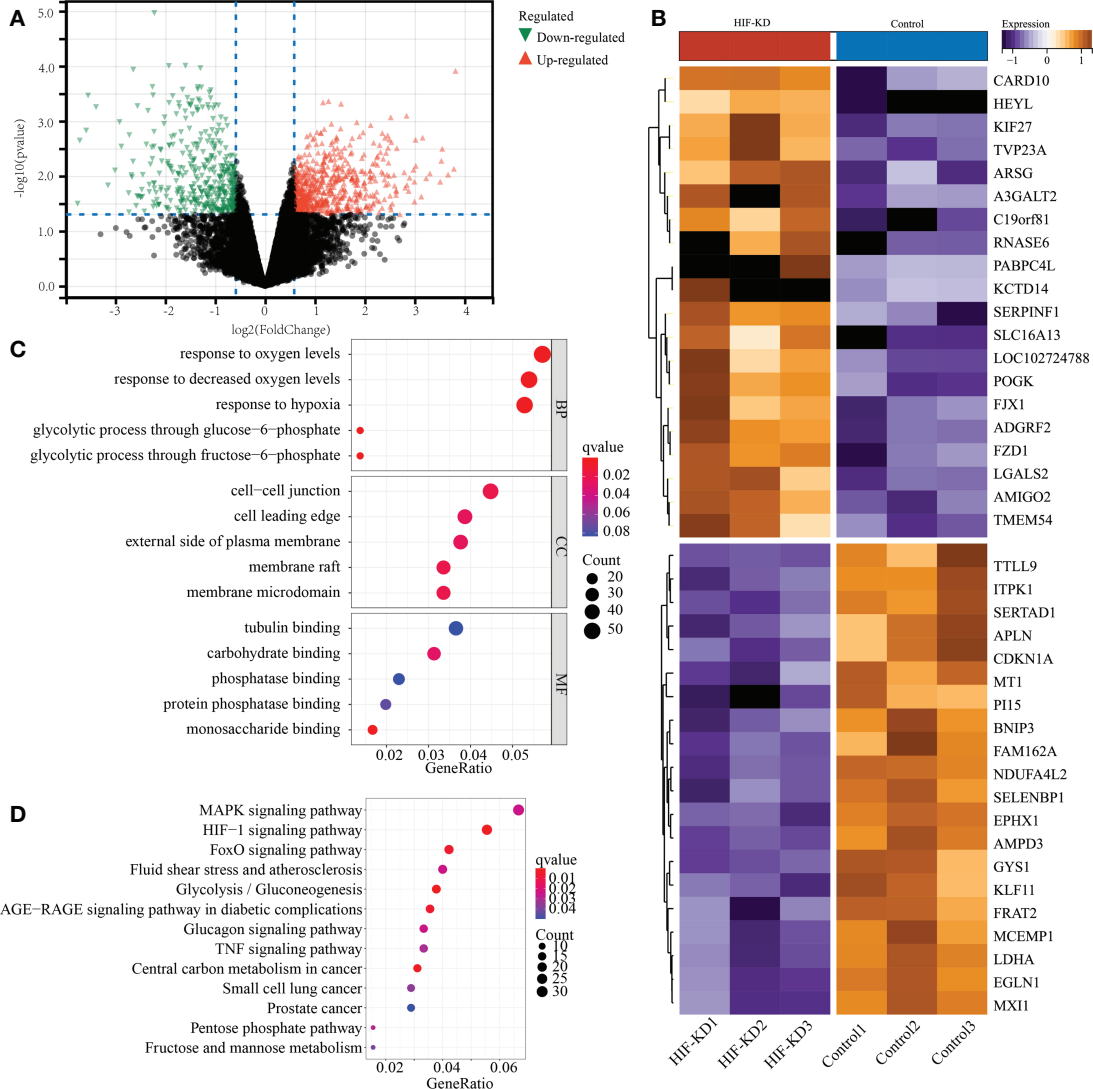
Functional validation of HIF1a gene in MC38 cell lines of mouse colon adenocarcinoma. **(A)** The volcano plot represents the differentially expressed genes between HIF1a-KD (knock down) and control cell lines. **(B)** The top 20 differentially expressed genes were shown in the heatmap. **(C)** GO anlysis of differentially expressed genes. **(D)** KEGG analysis of differentially expressed genes.

We obtained the raw data of transcriptome sequencing after knocking down HIF1α gene, including 16941 gene expression of 3 paired MC38 cell lines. Compared with control cell lines, knock-down of HIF1α gene in MC38 cell lines showed extremely distinct molecular characteristics. A total of 1074 DEGs were identified, with 660 up-regulated genes and 414 down-regulated genes (**Figure 12A**). The top 20 DEGs were shown in the heatmap form (**Figure 12B**). GO and KEGG analysis indicated that these DEGs were mainly enriched in the following terms: regulation of oxygen levels, NAD metabolic process, pyruvate metabolic process, cell−cell junction, GTPase regulator activity, MAPK signaling pathway, HIF−1 signaling pathway, FoxO signaling pathway, and glycolysis (**Figures 12C, D**). Overall, these findings validated the regulatory role of HIF1α in metabolism reprogramming of COAD.

## Discussion

Colon adenocarcinoma is the primary cause of cancer-related mortalities in humans, and its onset and progression are influenced by the presence and activity of sphingolipid components in tumors (57, 58). According to reports, sphingolipid is a bioactive molecule that plays a crucial regulatory function in the majority of body cells, including cell development control, proliferation, and programmed cell death, as well as the regulation of exosome production (59, 60). As demonstrated in the studies, the majority of the enzymes engaged in sphingolipid metabolism are responsible for the development of colon cancer (61, 62). Accumulating evidence has suggested that ceramide and sphingosine 1-phosphate (S1P) served as the crucial contributor in the occurrence and progression of COAD. The process of SLP metabolism can be roughly divided into the following steps: sphingomyelin-ceramides-sphinganine-S1P-hexadecenal. Some SLP-metabolizing enzymes have been reported to be dysregulated in human colon cancers (63). Majority of the enzymes have the potential to increase the ratio of sphingosine-1-phosphate (S1P) to ceramide, which in turn promote colon cancer cell survival, proliferation and cancer progression (63, 64). Colon cancer is associated with a reduction in alkaline-sphingomyelinase activity, which in turn decreases the hydrolysis of sphingomyelin and production of ceramide (65). The absence of ceramide in colon cancer may contribute to its growth because of its ability to suppress the proliferation of tumor cells (66). In addition, the phosphorylation of sphingosine by sphingosine kinases 1 (SK1) results in the formation of S1P, which promotes the growth of colon cancer cells. Studies on human colon cancer specimens have shown that the expression level of SK1 was significantly higher in COAD than normal mucosa (67). During metastasis, the expression of SK1 steadily rises (68). Collectively, disorders of enzyme activities contribute to the aberrant expression of substrates and products of SLP metabolism, and further promote the occurrence and progression of COAD. In addition, the aberrant gene expressions involved in sphingolipid metabolism may contribute to colon cancer growth and influence the treatment response of colon cancers as well. Additional research into SLP metabolism may offer a new therapeutic target for colon cancer (58).

Animal research and clinical observations have shown that the SLP metabolism of colon cancer cells has altered dramatically (66). To determine the extent to which SLP metabolism influences the prognosis of COAD patients and which genes influence the course of COAD, further research is required. In this work, we began by identifying the genes of SLP metabolism that exhibited a strong link to the prognosis of individuals suffering from COAD. Then, a pan-cancer study of these genes was conducted to highlight the significant impact of genes associated with SLP metabolism on a range of human malignancies. Based on the SLP metabolic genes, an unsupervised cluster analysis was carried out, and a prognostic risk score model was developed for estimating the probability of survival among patients with COAD.

Studies have shown that sphingolipids and enzymes that comprise the S1P pathway influence the development of ovarian cancer (69). Moreover, SLP metabolism affects the amount of estrogen in the body, thereby influencing the onset and progression of prostate cancer; however, the precise mechanism has not been elucidated. In our investigation, we chose sphingolipid metabolic genes for pan-cancer analysis, and the findings indicated that the BIRC5 protein was expressed at a high level in most of the malignancies, consistent with earlier research (38, 70, 71). BIRC5 is a tiny, multi-subtype protein whose role is to suppress apoptosis and promote cell growth (72). Additionally, reduced NGFR expression was observed in COAD. NGFR, a member of the tumor necrosis factor receptor family, is a multifunctional cell surface receptor that stimulates cell survival and differentiation during neural development, among other tasks (73). Yang and colleagues have shown that the NGFR expression is mainly down-regulated in colorectal cancer (55). NGFR inhibits tumor cell proliferation, migration, and invasion by inducing apoptosis and G1 phase arrest, which is useful in inhibiting the onset and advancement of tumors (55). More importantly, through the activation of S100A9, NGFR enhances the chemosensitivity of colorectal cancer cells by promoting the apoptotic and autophagic effects of 5-fluorouracil. Consistent with our result, the deletion of NGFR protein in colorectal cancer is linked to a poor OS in people suffering from colorectal cancer, which is an independent predictor of the prognosis of colorectal cancer (74).

Sphingolipids play a crucial role in the plasma membrane and have been shown to modulate the activity of surface receptors on immune cell surfaces (75). These metabolites have a role in the secretion of bioactive mediators such as S1P and ceramides, which control the crucial pathways necessary for the activation of immune cells and affect lymphocyte efflux and migration into the tumour microenvironment (TME) (76). Ceramide promoted the tumor-associated macrophages (TAMs) to differentiate into M1-like macrophages through activation of the protein kinase C pathway (77). Following this, M1-like macrophages induced tumor death *via* secreting IL-6, IL-10, IL-12, and TNF-α (75). Ceramide-mediated alterations of the rate of T helper 1 cell (Th1) and Th2 in host-TME led to TME skewed toward proinflammatory milieu, and then inhibit tumor growth (75). Moreover, C2-ceramide exerts its anti-tumor activity by increasing the percentage of CD8+T cells and producing perforin and granzyme B (77). However, S1P enhanced the expression of Bcl-2 in macrophages and encouraged the polarization of M2-like macrophages, which further facilitated tumor evasion (78, 79). Collectively, SLP metabolism is intimately associated with tumour immunity, and changing levels of sphingolipid metabolism are crucial determinants regulating TME. Similarly, our findings revealed that PD-1, IL-10, and chemokine signal pathways are among the several immune-related pathways that are substantially connected with these SLP genes. Overall, our findings indicate that individuals with colon adenocarcinoma have an aberrant SLP process and that the metabolic reprogramming of tumors is intimately tied to their immunological microenvironment. The pan-cancer description of the SLP genes provides a good framework for future research into additional malignancies.

Consequently, based on genes associated with sphingolipid metabolism, 1008 individuals with COAD were effectively classified into two metabolic subgroups (C1 and C2 subtypes). Patients with subtype C1 had an active sphingolipid metabolism pathway and relatively high levels of sphingolipid metabolism genes, but their prognoses were poor. These findings showed that the sphingolipid metabolism might have a detrimental role in the pathophysiology of colon adenocarcinoma, and that focused intervention of the sphingolipid metabolic pathway might improve the clinical outcomes of COAD patients. More importantly, we discovered that the NOTCH signaling pathway, TGF-β signaling pathway, apoptosis, angiogenesis, and hypoxia pathways were considerably active in the C1 subtype, which might be one of the causes of the subtype’s poor prognosis. The NOTCH signaling system is a highly conserved developmental pathway essential for apoptosis, tissue structure, and morphogenesis (80, 81). Increasing data suggest that this pathway is aberrant in several types of malignant tumors and may regulate oncogenes or tumor suppressors (82). Staudacher et al. revealed that the TGF-β signaling pathway is strongly linked to the incidence and progression of advanced colon cancer (83).

Considering that the process of metabolic reprogramming in tumors may be accompanied by an immune microenvironment imbalance, we investigated the possible variations in the immune microenvironment between C1 and C2 metabolic subtypes in depth. Intriguingly, the C1 subtype with active sphingolipid metabolism is coupled by strong infiltration of cells of the immune system and high expression levels of the immune checkpoints. Specifically, the infiltration abundance of B cells, CD4+T cells, CD8+T cells, and macrophages was greater in the C1 subgroup than that in the C2 subgroup, and immune checkpoint-associated genes (such as PDCD1LG2, TIGIT, TNFSF4, LAG3, CD86, CD40, and CD48) were up-regulated in C1 subgroup. Masugi et al. found that the PDCD1LG2 expression in colorectal cancer tumors was inversely connected with Crohn’s lymphoid response, indicating that the PDCD1LG2 might prevent the formation of tertiary lymphoid tissue in colorectal cancer (84). Furthermore, by studying the evolutionary genetic algorithms, Alderdice and colleagues found that IL2RB is a potential predictive biomarker of immune checkpoint therapy for colorectal cancer, which is consistent with our study (85). Immune checkpoint inhibitors are a new method of immunotherapy, which does not simply refer to improving the immunity of the body, but to improving the immune microenvironment around the tumor, thereby activating the activity of immune cells in the body to achieve the purpose of anti-tumor (86). The current immune checkpoint inhibitor treatment method has set off a boom in many tumors and has also achieved significant clinical progress, including lung cancer, lymphoma, melanoma, bowel cancer, and multiple other tumor treatments (87). The findings of this research provide a fresh approach to the future therapy of immunological checkpoints for colon cancer.

Finally, we assessed the ability of genes involved in sphingolipid metabolism to predict COAD survival rate. The findings demonstrated that patients with high risk had considerably lower survival rates than those with low risk. The AUC value of our prognostic model shows excellent diagnostic performance in the training set, internal verification set, and external verification set. In conclusion, the findings of this study revealed that the prognostic model based on sphingolipid metabolism might reliably predict the likelihood of survival for COAD patients. Our predictive diagnostic model comprises ten genes associated with sphingolipid metabolism, including TCEAL2, GRP, EEF1A2, LSAMP, C2CD4A, RBP1, ANO1, HEYL, IGF1, and LAMA2. Taglia et al. have reported that GRP/GRPR is responsible for promoting the binding of CD16+ and CD94+ natural killer cells following inducing expression of Hsp72 by signaling *via* focal adhesion kinase, thus contributing to tumor cell cytolysis (88). EEF1A2 served as an epithelial-mesenchymal transition-related gene that was found to be closely linked to the clinical outcomes of colon cancer, which was consistent with the outcomes of our study. Zhou et al. demonstrated that reduced expression of TCEAL2 may be associated with renal cell carcinoma carcinogenesis (89). Furthermore, Chang et al. demonstrated that LSMAP, a tumor suppressor, influences the incidence and progression of lung cancer by regulating the epithelial-mesenchymal transition pathway (90). C2CD4A is a possible diagnostic marker for colon cancer, and its expression is strongly associated with the tumor stage of colon cancer, which is almost compatible with our results (91). As an intracellular molecular chaperone, RBP1 is highly down-regulated in most malignancies in humans, such as breast cancer (92). Jiang et al. demonstrated that the expression of ANO1 is associated with advanced colon cancer lymphatic metastasis (93). The epidermal growth factor receptor/extracellular signal-regulated kinase signal pathway activation influences the onset and progression of colon cancer by upregulating the expression level of ANO1 in colon cancer (93). Weber et al. reported that the overexpression of HEYL might suppress the formation and migration of tumor cells following inhibiting the intravasation of metastasis-initiating cells (94). Gao et al. have reported that IGF1 was associated with the pivotal precursor to colorectal cancer (95). Lee et al. uncovered that the methylation level of LAMA2 had a crucial role in the onset and advancement of colorectal cancer (96). Although the functions of these hub genes in COAD need additional investigation, our analysis reveals that they are key prognostic variables and may be possible treatment targets.

There are still various significant limitations that need additional investigation. Initially, we simply investigated the prognostic significance of SLP genes in COAD. Due to the limitation of RNA-sequencing, this study presents genes that encode enzymes that are mainly transcription factors and growth factors that are indirectly involved in the metabolism of sphingolipids, such as EEF1A2, GRP, HESR, IGF1, LAMA2, etc. However, the expression of certain genes such as SMPD1, SMPD3, SGMS1, SGMS2, ASAH1, ASAH2, etc, that encode enzymes directly related to the SLP metabolism was not detected, and some genes were not associated with the clinical outcomes of COAD. More research based on new sequencing technologies are required to uncover the potential contributions of genes that encode enzymes directly related to the SLP metabolism in COAD. Secondly, even though our analysis confirmed the varied expression levels of genes related to the risk model at the mRNA as well as the protein levels, this research would become more comprehensive if in-depth biological experiments are further conducted. Consequently, more well-designed experimentation is required to confirm our results. Nevertheless, despite the aforementioned limitations, it is undeniable that this study made the first thorough investigation of SLP genes in COAD and summarization of the pan-cancer overview of SLP genes. Additionally, the cluster and risk score may be used independently to assess the prognosis in people suffering from COAD.

## Conclusion

In our investigation, we examined 26 SLP genes strongly linked to the prognosis of COAD patients. Pan-cancer characterization of SLP genes highlighted their crucial role in the onset and progression of tumors. Individuals suffering from COAD were effectively sorted into two metabolic subgroups as per the features of SLP gene expression. Active sphingolipid metabolism subtypes were associated with the unbalanced local tumor immune microenvironment and poor clinical outcomes. Based on the metabolic classifier, we developed a unique and reliable prognostic model for patients with COAD, which gives a new perspective on the prognostic assessment of cancer patients.

## Data availability statement

The original contributions presented in the study are included in the article/**Supplementary Material**. Further inquiries can be directed to the corresponding author.

## Author contributions

QY, WZ, and WS contributed to this study equally. All the authors participated in the design of study, data collection and processing, bioinformatics analysis, and writing and revising the manusript. All authors read and approved the final manuscript. All authors contributed to the article and approved the submitted version.

## Acknowledgments

We thank Bullet Edits Limited for the linguistic editing of the manuscript.

## Conflict of interest

The authors declare that the research was conducted in the absence of any commercial or financial relationships that could be construed as a potential conflict of interest.

## Publisher’s note

All claims expressed in this article are solely those of the authors and do not necessarily represent those of their affiliated organizations, or those of the publisher, the editors and the reviewers. Any product that may be evaluated in this article, or claim that may be made by its manufacturer, is not guaranteed or endorsed by the publisher.
